# Polypharmacy and trajectories of health-related quality of life in older adults: an Australian cohort study

**DOI:** 10.1007/s11136-022-03136-9

**Published:** 2022-04-27

**Authors:** Muhamad S. Aljeaidi, Miriam L. Haaksma, Edwin C. K. Tan

**Affiliations:** 1 Medical School, The University of Western Australia, Perth, WA Australia; 2 Department of Public Health and Primary Care, Leiden University Medical Center, Leiden, The Netherlands; 3 Faculty of Medicine and Health, School of Pharmacy, The University of Sydney, Pharmacy Building A15, Science Road, Camperdown, Sydney, NSW 2006 Australia

**Keywords:** Polypharmacy, Quality of life, Aged, Ageing, Longitudinal studies

## Abstract

**Background:**

Health-related quality of life (HRQoL) is an important outcome measure when considering medical treatment; however, the impact of polypharmacy on trajectories of HRQoL over time is unknown. This study aimed to investigate the association between polypharmacy status and trajectories of HRQoL in older adults.

**Methods:**

A longitudinal cohort study of 2181 community-dwelling adults, 65 years and older, who participated in the 2013 to 2017 waves of the Household Income and Labour Dynamics in Australia (HILDA) Survey. Polypharmacy was defined as the regular use of ≥ 5 prescription medications. Polypharmacy status was categorised into no polypharmacy, in 2013 only (baseline only polypharmacy), in 2017 only (incident polypharmacy) or at both time points (persistent polypharmacy). HRQoL was assessed through the SF-36 questionnaire generating two summary scores: physical component summary (PCS) and mental component summary (MCS). Linear mixed-effects models stratified according to polypharmacy status and change in comorbidities were used to assess trajectories of HRQoL.

**Results:**

Older adults with persistent polypharmacy had lowest scores for HRQoL measures from 2013 to 2017. After adjusting for all covariates, those with incident polypharmacy had the steepest annual decline in both the PCS and MCS: − 0.86 in PCS and − 0.76 in MCS for those with decreasing or stable comorbidities, and − 1.20 in PCS and − 0.75 in MCS for those with increasing comorbidities.

**Conclusions:**

Polypharmacy was associated with poorer HRQoL, even after adjusting for confounders. Incident polypharmacy was found to be associated with a clinically important decline in HRQoL and this should be considered when prescribing additional medication to older adults.

**Supplementary Information:**

The online version contains supplementary material available at 10.1007/s11136-022-03136-9.

## Plain English summary

Older adults are often prescribed multiple medications to manage multiple health conditions. When an individual is taking five or more medications simultaneously, they are considered to be on polypharmacy. There is a growing concern about polypharmacy, particularly in older adults, due to its association with undesirable health outcomes. However, it is unclear how polypharmacy status may impact the overall well-being or quality of life of older adults over time. In this study, we explored the quality of life of older Australians aged 65 years and older. The participants answered a detailed questionnaire annually from 2013 to 2017. The results from this study indicate that greater exposure to polypharmacy may be associated with reduced quality of life and this was deemed to be clinically important. Moreover, those who were later exposed to polypharmacy during the study period were found to have the sharpest decline in their quality of life over time.

## Introduction

Multimorbidity is common in older adults leading to the concurrent use of multiple medications or polypharmacy [1, 2]. Polypharmacy is commonly defined as the use of five or more regular medications [3–5] and is inherently dynamic depending on the health status of the patient [4]. A recent study in Australia found that the most commonly used types of medications in older adults with polypharmacy were medications for the cardiovascular system followed by alimentary tract and metabolism, and the nervous system [4]. Polypharmacy may be a necessity when managing multimorbidity [5]; however, it has been associated with negative impacts on health especially in vulnerable older adults [5]. Polypharmacy has been linked to multiple undesirable health outcomes including falls, hospitalisation, and mortality [5–7]. Polypharmacy may be the result of a disease-centred approach with clinicians adhering to multiple, concurrent disease-specific guidelines [8, 9].

Person-centred care is gaining traction as the treatment approach for all individuals, including older adults with multiple chronic conditions [8]. Such an approach is focused on achieving patients’ specific health outcomes as it involves the patient in the decision-making process, aligning medical interventions with what matters most to them [8, 9]. This can include assessing health-related quality of life (HRQoL). The concept of HRQoL is often described as a measure of the individual’s perception of life taking into consideration different aspects of their health including physical and mental health, cognitive and emotional status, and overall general health [10]. HRQoL has been identified as one of the most important outcome measures for patients when considering medical treatment including pharmacological therapy [6, 11–13]. Multiple factors that are linked to polypharmacy initiation or consequence, including age, comorbidities, physical and cognitive functional level, social status, education, and standard of living, have also been reported to influence HRQoL [14–20]

Despite this, the impact of polypharmacy on HRQoL receives little attention. As both polypharmacy status and HRQoL may change over time, especially in older adults, longitudinal studies with adequate follow-up periods are needed to assess this association. Results from previous research, which has been limited to cross-sectional studies and specific subgroups, have been inconsistent [14, 21–25]. Thus, the aim of this study was to investigate the association between polypharmacy status and trajectories of HRQoL in community-dwelling older adults. We hypothesised that greater exposure to polypharmacy would be associated with poorer HRQoL compared to those who never had polypharmacy.

## Methods

### Study population

This cohort study was based on data from the Household Income and Labour Dynamics in Australia (HILDA) Survey. In the present study, HRQoL was assessed annually from 2013 to 2017, and polypharmacy status was assessed in 2013 and 2017. Briefly, the HILDA survey is one of the largest longitudinal surveys in Australia that collects information from community-dwelling participants covering a wide range of dimensions from family relationships and finance to health and education. Further details about the survey and its methodology have been reported elsewhere [26]. The sample population included in the present study were older adults aged 65 years and over who participated in 2013, had medication history recorded, and complete information on covariates at baseline and at follow-up. Participants who do not satisfy any of these criteria were excluded.

### Study sample

**Fig. 1:**
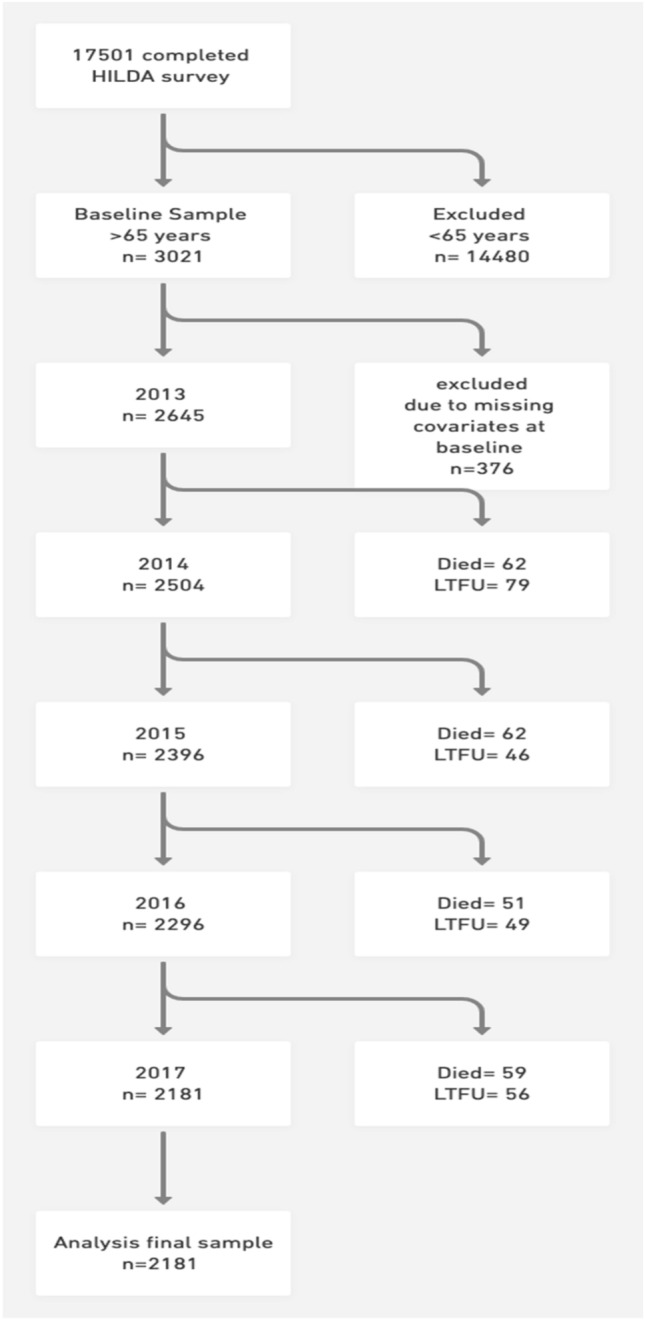
Flow chart of the study participants

A total of 17,501 participants completed wave 13. Of these, 3021 (17.3%) were aged 65 years and older and identified as potential participants. Of these, 2645 (87.6%) had complete information on covariates. Those who were excluded due to incomplete information at baseline (*n* = 376) were older, less educated, less likely to be married, and more socially active. Of the initial 2645, 464 were excluded due to missing covariates at follow-up. Overall, 2181 participants fulfilled the inclusion criteria and were included in the final analysis. Figure 1 shows a flow chart of the study.

### Polypharmacy status

In Australia, the Pharmaceutical Benefits Scheme (PBS) provides universal health care that allows all citizens and permanent residents to access a range of subsidised healthcare services including subsidised medicines [27]. In the present study, the use of regular prescription medications was measured in 2013 and 2017 only, and this was obtained from responses to the question: “All together, how many prescription medications do you take on a regular basis?”. In this study, polypharmacy was defined as the concurrent use of five or more regular prescription medications [3]. Participants with polypharmacy at neither baseline nor follow-up were classified as ‘no polypharmacy’; those with polypharmacy in 2013 only were classified as ‘baseline only polypharmacy’; those with polypharmacy in 2017 only were classified as ‘incident polypharmacy’; and those with polypharmacy at both time points were classified as ‘persistent polypharmacy’.

### Health-related quality of life (HRQoL)

HRQoL was assessed in HILDA through the Medical Outcomes Study Short Form (SF-36). The SF-36 is one of the most widely used measures of HRQoL [28]. Detailed description of SF-36 has been explained elsewhere [29]. Briefly, it is a self-completed questionnaire comprising 36 items that cover eight domains including general health, pain, vitality, social, mental and physical function, and role limitation due to emotional or physical health. Using factor analysis of the eight scales, two summary scores of health known as the physical component summary (PCS) and the mental component summary (MCS) were generated. Traditionally, scores from all domains are combined and transformed to generate PCS and MCS. However, as explained by Dockery et al. this may lead to inaccurate results in older adults [30]; for instance, in the factor analysis, the physical function domains negatively correlate with mental health, and hence, MCS scores would increase, not necessarily due to improvement of mental health but rather because of deterioration of physical function [30]. Therefore, in the present study, PCS scores were calculated from the four subscales that were positively correlated with physical function in the factor analysis. Those subscales were role-physical, bodily pain, physical functioning and general health. Similarly, MCS scores were calculated from the four subscales that were positively correlated to mental health in the factor analysis. Those subscales included vitality, social functioning, role limitations and mental health. A higher score (ranging from 0 to 100) on a component reflects a better HRQoL [29]. The SF-36 scores reported in HILDA have been previously validated to support their use as general measures of physical and mental health status in the Australian population [28].

### Covariates

Covariates included age, gender, education level, marital status, comorbidities, socio-economic status, and lifestyle behaviours at baseline (2013). Education was dichotomised into high school (i.e. 12 years of education) or higher vs less than high school. Marital status was dichotomised into ‘married/defacto’ vs ‘single/separated/divorced/widowed’. Number of comorbidities was obtained from questions that assessed the presence of a diagnosis of arthritis, asthma, any type of cancer, chronic bronchitis and/or emphysema, depression and/or anxiety, type 1 diabetes, type 2 diabetes, hypertension, heart disease, or other serious circulatory condition, e.g. stroke. These comorbidities were included as they are likely to impact HRQoL [31]. Change in comorbidities was calculated as the difference in number of comorbidities between 2013 and 2017, and categorised as ‘stable/decrease’ and ‘increase’. Socio-economic Index For Areas (SEIFA) is a relative measure of socio-economic advantage and disadvantage of areas in Australia provided by the Australian Bureau of Statistics [32]. SEIFA has multiple indices; in HILDA, the Index of Relative Socio-Economic Advantage and Disadvantage (IRSAD) was used [26]. Detailed explanation of SEIFA and its indices is reported elsewhere [32]. Briefly, IRSAD provides a summary measure of relative socio-economic advantages and disadvantages experienced, on average, by individuals living in that area [32]. For example, an area with majority of the households with low income or unskilled occupations, and few households with high income or skilled occupations would generally have a low score indicating a relatively greater disadvantage and a lack of advantage [32]. We treated SEIFA-IRSAD deciles as a continuous variable ranking areas from 1 (most disadvantaged) to 10 (most advantaged). Physical activity was measured as the number of days participants engaged in moderate or intensive physical activity for at least 30 minutes. This was included in the analysis as a dichotomous variable: ‘3 days a week or more’ or ‘less than 3 days a week’. Social life was measured by asking participants how often they get together socially with friends or relatives not living with them, and was dichotomised into ‘once a month or less’ or ‘more than once a month’. Frequency of alcohol intake was dichotomised into: drinking on ‘less than 5 days a week’ or ‘5 or more days a week’. Lastly, smoking was dichotomised into ‘smoker’ or ‘non-smoker’.

### Statistical analysis

Baseline characteristics were described according to polypharmacy status and reported as means and standard deviations (SDs), or frequencies and percentages, as appropriate. Linear mixed-effects models were used to model the change in PCS and MCS scores from 2013 to 2017. Models were stratified according to polypharmacy status (none/baseline only/incident/prevalent) and change in comorbidities (stable/decrease vs. increase), resulting in 8 strata. The final models included intercept and slope (time) as random effects. Variances of the residuals and random effects were assumed to be uncorrelated. Allowing the random intercept and random slope to be correlated resulted in non-convergence of the models. For the stratum ‘baseline only polypharmacy and increased comorbidities’, the model contained a random intercept only due to the small sample size of this stratum. Time was based on the exact date of interviews. A quadratic slope was tested but was not significant. Hence, HRQoL trajectories were modelled as a linear function of time in years with 2013 as time = 0. Assumptions of linearity, homoscedasticity and normality of residuals were confirmed by visual inspection of graphs. The main analysis was adjusted for baseline comorbidities, education, marital status, SEIFA decile, social life, physical activity, smoking and alcohol intake. All covariates were mean centred and included as intercept and slope predictors. The results of these models were reported as estimated fixed effects (β value) and 95% confidence intervals (CIs). To assess the impact of different definitions of polypharmacy on the observed results, we conducted a sensitivity analysis in the incident polypharmacy model using different cut offs for polypharmacy (i.e. defining polypharmacy as ≥ 3, ≥ 4, ≥ 6, and ≥ 7 medications). All analyses were conducted using the Statistical Package for the Social Sciences (SPSS) version 25 (IBM Corp Armonk, NY) and GraphPad Prism 9.2.0 for Windows, GraphPad Software, San Diego, California USA.

## Results

Overall, the study included 2181 participants at baseline, with a mean age of 72.8 (SD 6.3) years and 54.8% were female (Table 1). The majority of the sample did not have polypharmacy (61.9%), baseline only polypharmacy was present in 5.7%, incident polypharmacy in 11.2% and persistent polypharmacy in 21.1%. The average scores of PCS and MCS over the whole study period are reported in supplementary Table S1. The prevalence of different chronic diseases at baseline is reported in Table S2.

**Table 1:** Baseline characteristics of participants by polypharmacy status

	Polypharmacy status				
	Overall (*n* = 2181)	No polypharmacy (*n* = 1351, 61.9%)	Baseline only polypharmacy (*n* = 125, 5.7%)	Incident polypharmacy (*n* = 245, 11.2%)	Persistent polypharmacy (*n* = 460, 21.1%)
Demographics					
Age, mean (SD), years	72.8 (6.3)	71.9 (6.0)	74.0 (6.5)	74.2 (6.8)	74.2 (6.5)
Female, *n* (%)	1195 (54.8)	740 (54.8)	75 (60.0)	127 (51.8)	253 (55.0)
High school education or above, *n* (%)	1176 (53.9)	771 (57.1)	59 (47.2)	129 (52.7)	217 (47.1)
Comorbidities at 2013, mean (SD)	1.66 (1.3)	1.15 (1.0)	2.14 (1.0)	1.78 (1.1)	2.96 (1.4)
Comorbidities at 2017, mean (SD)	1.88 (1.4)	1.33 (1.0)	2.13 (1.1)	2.37 (1.2)	3.15 (1.5)
Medications, mean (SD)	3.5 (3.1)	1.8 (1.3)	7.0 (2.8)	3.0 (1.2)	7.5 (2.8)
Married, *n* (%)	1365 (62.6)	888 (65.7)	71 (56.8)	143 (58.4)	263 (57.2)
Socialises > once a month. *n* (%)	1791 (82.1)	1132 (83.8)	93 (74.4)	197 (80.4)	369 (80.2)
SEIFA- IRSAD, mean (SD)	5.4 (3.0)	5.6 (3.0)	4.8 (2.7)	5.2 (3.0)	4.7 (2.8)
Physical activity ≥ 3 times a week, *n* (%)	1090 (50.0)	781 (57.8)	50 (40.0)	118 (48.2)	141 (30.7)
Smoking, *n* (%)	131 (6.0)	73 (5.4)	7 (5.6)	21 (8.6)	30 (6.5)
Alcohol intake ≥ 5 days per week, *n* (%)	574 (26.3)	374 (27.7	30 (24.0)	67 (27.3)	102 (22.2)
QoL summary measures					
PCS	43.8 (12.7)	48.1 (10.6)	38.6 (11.6)	41.3 (11.9)	33.3 (12.1)
MCS	50.8 (11.5)	54.1 (9.7)	47.1 (11.8)	49.3 (10.5)	42.8 (12.6)
QoL measures, mean (SD)					
General health	63.94 (21.6)	71.4 (17.9)	57.8 (21.0)	59.2 (19.4)	46.2 (21.0)
Mental health	78.5 (16.2)	81.8 (14.5)	75.2 (16.7)	77.6 (14.6)	70.4 (18.2)
Physical functioning	68.3 (25.5)	76.5 (21.3)	59.2 (24.0)	64.1 (24.1)	48.5 (25.8)
Social functioning	80.4 (24.5)	86.8 (20.6)	74.7 (25.5)	77.6 (23.0)	64.9 (27.9)
Vitality	61.2 (20.0)	67.0 (17.7)	55.2 (19.2)	58.2 (18.4)	47.4 (19.7)
Bodily pain	63.8 (25.0)	70.4 (22.2)	56.5 (24.9)	60.6 (24.2)	47.9 (25.3)
Role-physical	60.4 (43.3)	72.7 (38.7)	43.3 (42.4)	53.2 (43.8)	32.5 (40.4)
Role-emotional	80.1 (35.7)	87.0 (29.2)	69.1 (41.2)	78.2 (36.9)	63.9 (43.8)

*SEIFA-IRSAD* Socio-Economic Indexes for Areas-Index of Relative Socio-Economic Advantage and Disadvantage, *SD* standard deviation, *QoL* quality of life, *PCS* Physical component summary, *MCS* Mental component summary

**Fig. 2:**
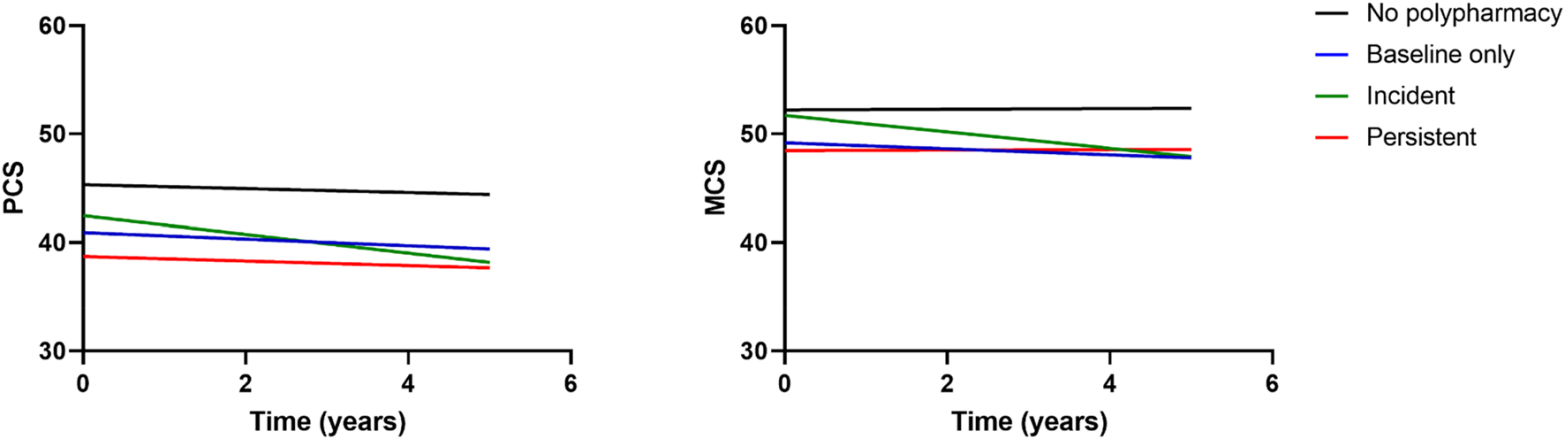
Quality of life trajectories in older adults with stable/decrease in comorbidities by polypharmacy strata. *PCS* physical component summary, *MCS* mental component summary

At baseline, both PCS and MCS were highest in the group without polypharmacy and lowest for the group with persistent polypharmacy in those with stable/decrease in comorbidities in the fully adjusted models (Fig. 2). The steepest decline in SF-36 components was seen in the incident polypharmacy group: − 0.86 in PCS and − 0.76 in MCS (Tables 2 and 3). Over the 4-year study period, this equated to a decline of − 3.44 in PCS and − 3.04 in MCS. Those without polypharmacy were more homogenous than other polypharmacy groups, based on the sizes of the random effects.

**Table 2:** Parameter estimates of mixed models for mental component summary (MCS) fitted in four polypharmacy strata for people with stable/decrease in comorbidities over four years

	No polypharmacy *N* = 914			Baseline only polypharmacy *N* = 87			Incident polypharmacy *N* = 130			Persistent polypharmacy *N* = 280		
	*β* (SE)	*P* value	95% CI	*β* (SE)	*P* value	95% CI	*β* (SE)	*P* value	95% CI	*β* (SE)	*P* value	95% CI
Fixed effects												
Intercept	52.2 (0.3)	< 0.001	51.6, 52.8	49.2 (1.5)	< 0.001	46.2, 52.2	51.7 (0.8)	< 0.001	50.2, 53.3	48.5 (1.0)	< 0.001	46.6, 50.4
Slope (per year)	0.03 (0.06)	0.645	− 0.1, 0.2	− 0.3 (0.2)	0.257	− 0.8, 0.2	− 0.8 (0.2)	< 0.001	− 1.1, − 0.4	0.02 (0.2)	0.912	− 0.4, 0.4
Random effects												
Variance in intercept	48.3 (2.6)	< 0.001	43.4, 53.7	105.8 (17.9)	< 0.001	75.9, 147.4	53.3 (8.1)	< 0.001	39.6, 71.7	82.1 (7.9)	< 0.001	68.0, 99.2
Variance in slope	0.3 (0.1)	0.006	0.2, 0.7	0.3 (0.6)	0.682	0.002, 30.7	1.6 (0.6)	0.005	0.8, 3.1	0.7 (0.4)	0.061	0.2, 2.0

Intercepts and slopes for all models are adjusted for age, sex, education, social life, marital status, Socio-Economic Indexes for Areas decile, physical activity, smoking and alcohol intake, and baseline comorbidities; All covariates were mean centred and included as intercept and slope predictors; MCS: Mental component summary; Polypharmacy defined as ≥ 5 medications. Covariate estimates are reported in Table S10

**Table 3:** Parameter estimates of mixed models for physical component summary (PCS) fitted in four polypharmacy strata for people with stable/decrease in comorbidities over four years

	No polypharmacy *N* = 913			Baseline only polypharmacy *N* = 87			Incident polypharmacy *N* = 130			Persistent polypharmacy *N* = 280		
	*β* (SE)	*P* value	95% CI	*β* (SE)	*P* value	95% CI	*β* (SE)	*P* value	95% CI	*β* (SE)	*P* value	95% CI
Fixed effects												
Intercept	45.3 (0.3)	< 0.001	44.7, 46.0	40.9 (1.3)	< 0.001	38.3, 43.6	42.5 (1.0)	< 0.001	40.6, 44.3	38.7 (0.9)	< 0.001	37.0, 40.5
Slope (per year)	− 0.2 (0.1)	0.009	− 0.3, − 0.1	− 0.3 (0.2)	0.177	− 0.7, 0.1	− 0.9 (0.2)	< 0.001	− 1.3, − 0.5	− 0.2 (0.2)	0.214	− 0.5, 0.1
Random effects												
Variance in intercept	55.8 (3.03)	< 0.001	50.1, 62.0	84.9 (14.1)	< 0.001	61.4, 117.5	83.2 (12.0)	< 0.001	62.7, 110.3	70.5 (6.6)	< 0.001	58.6, 84.8
Variance in slope	0.7 (0.2)	< 0.001	0.5, 1.1	0.18 (0.5)	0.696	0.001, 27.8	2.01 (0.6)	0.001	1.1, 3.7	0.4 (0.3)	0.238	0.1, 1.7

Intercepts and slopes for all models are adjusted for age, sex, education, social life, marital status, Socio-Economic Indexes for Areas decile, physical activity, smoking and alcohol intake, and baseline comorbidities; All covariates were mean centred and included as intercept and slope predictors; PCS: physical component summary; Polypharmacy defined as ≥ 5 medications. Covariate estimates are reported in Table S11

Similarly, the steepest decline for those who had an increase in comorbidities was seen in the incident polypharmacy group: − 1.20 in PCS and − 0.75 in MCS, equating to a decline of − 4.80 in PCS and − 3.00 in MCS over the four years of the study period (Figure S1; Tables S3 and S4). The age and sex adjusted models are reported in Tables S5, S6, S7, S8. The results obtained from the sensitivity analysis, which examined different polypharmacy cut offs in the incident polypharmacy model, showed similar findings (Supplementary Tables S9.1–4).

## Discussion

To our knowledge, this national study of older adults is the first to investigate trajectories of HRQoL by polypharmacy status. It found that greater exposure to polypharmacy was associated with poorer HRQoL. Moreover, incident users of polypharmacy showed a significantly steeper decline in HRQoL over time compared to people who were not exposed to polypharmacy.

Our finding that polypharmacy was associated with a lower HRQoL is in line with previous research [14, 21, 33, 34]. This is plausible as polypharmacy not only reflects multimorbidity and disease severity, but also increased risk of drug-drug and drug-disease interactions, adverse drug events, and inappropriate prescribing. In contrast to our findings, Lalic et al. reported no association between polypharmacy (when defined as ≥ 9 regular medications) and staff informant-rated HRQoL in residents of aged care services [23]. These conflicting findings could be due to the different definitions of polypharmacy used and/or due to the different settings in which the studies were conducted.

The reason why incident polypharmacy was associated with a steeper decline in HRQoL needs further research. This association is noteworthy as polypharmacy incidence increases with age. In a prospective cohort study conducted in Sweden, authors noted that 20% of older adults who were free of polypharmacy at baseline, became exposed to polypharmacy during the next year [18]. In the present study, we found those who were exposed to polypharmacy at follow-up had a steeper decline in HRQoL compared to the other groups. In fact, over time those with incident polypharmacy developed low PCS and MCS scores that were comparable to those with persistent polypharmacy. This finding may be due to the patient's health worsening with onset of other comorbidities or worsening of existing comorbidities; however, the association remained significant even after we adjusted for change in comorbidities in our analysis. It may be plausible that the deterioration in PCS and MCS might have been less severe in a patient-centred and integrated care service; however, a previous study found receiving person-centred and integrated care had no clinical benefit in health and wellbeing of older adults living in the community after following them for 12 months [35]. The authors of that study suggested the duration of such an intervention may require a longer follow-up time to detect its benefits [35].

In the present study, over four years of follow-up we found a decline of − 3.44 in PCS and − 3.04 in MCS for those with incident polypharmacy with stable/decrease in comorbidities, and − 4.8 in PCS and − 3.0 in MCS for those with incident polypharmacy with increase in comorbidities. The minimally important difference (MID) refers to the smallest change in PCS or MCS scores that would lead to a clinically relevant change. For the general population, a MID of 2 points in PCS and 3 points in MCS has been reported [36]. These findings show that introducing polypharmacy is associated with a clinically relevant decline in the physical and mental components of HRQoL measures. Moreover, a previous US cohort study found that a 1-point lower score on some HRQoL measures, such as PCS scores, was associated with a 9% increased risk of mortality in patients with diabetes mellitus [37]. Future studies are needed to investigate the MID in HRQoL measures for older adults with chronic disease and polypharmacy.

Since older adults are reported to be at increased risk of adverse drug events from polypharmacy [38, 39], and considering the significant rate of decline in HRQoL seen in the present study, patients with incident polypharmacy may benefit from different treatment approaches [2]. This includes a more patient-centred approach with a regular assessment of polypharmacy appropriateness [40], and a more proactive treatment plan where patients, and possibly carers, are involved in a regular assessment of their current medications and HRQoL. This will help identify opportunities to intervene and may potentially facilitate trials of non-pharmacological interventions, and use of fewer medications [40]. Moreover, interventions that target polypharmacy should consider those with polypharmacy as well as those at risk of being exposed to it.

In addition, we also noted those with baseline only polypharmacy had a lower HRQoL when compared to those with no polypharmacy at baseline. Previous research has shown that interventions that reduce the number of medications do not necessarily lead to improved HRQoL [41]. Unlike other factors that may impact HRQoL, polypharmacy is potentially modifiable. Policy makers and the health system should support programmes that address polypharmacy such as those that incorporate structured medication reviews to optimise existing pharmacological and non-pharmacological therapies for older patients, for example, through allocating sufficient funding to those programmes [42, 43]. Furthermore, clinicians treating older adults should anticipate there may be a long-lasting influence of polypharmacy on HRQoL, and consider regular medication reviews as early as possible, where appropriate.

A major strength of the present study was the large nationally representative sample and the use of longitudinal data with a long follow-up time. In addition, to the best of our knowledge, this study was the first to look at trajectories of HRQoL by polypharmacy exposure status. Nevertheless, there were some limitations to note. Firstly, the HILDA survey only assessed the total number of prescription medications, and hence data on the use of complementary and over-the-counter medications were not accounted for in the present study. In addition, medication use was based on patient self-report which may introduce recall bias. Also, it was not possible to determine the level of adherence to medications from the data collected in HILDA. Furthermore, due to lack of information, it was not possible to consider the type of medications used by each participant and whether or not they were clinically appropriate. Another limitation to this study was the inability to adjust for the severity of comorbidities in the main analysis, so the HRQoL trajectories observed in our study might, in part, have resulted from changes in these factors. Lastly, another limitation to the study is the possible selection bias that may have resulted from the loss of follow-up either due to death or dropping out.

In this study of community-dwelling older adults, greater exposure to polypharmacy was associated with lower HRQoL. Moreover, incident polypharmacy was associated with a clinically relevant decline in HRQoL over time. Clinicians should carefully consider the impact introducing polypharmacy may have on a patients’ quality of life.

## Supplementary Information

Below is the link to the electronic supplementary material.

Supplementary file1 (DOCX 128 kb)

## Data Availability

The HILDA dataset used and analysed during the current study may be available upon request; for more information please visit: https://melbourneinstitute.unimelb.edu.au/hilda/for-data-users.

## References

[1] Page AT, Falster MO, Litchfield M, Pearson SA, Etherton-Beer C (2019). Polypharmacy among older Australians, 2006–2017: A population-based study. Medical Journal of Australia.

[2] Qato DM, Wilder J, Schumm LP, Gillet V, Alexander GC (2016). Changes in prescription and over-the-counter medication and dietary supplement use among older adults in the United States, 2005 vs 2011. JAMA Internal Medicine.

[3] Masnoon N, Shakib S, Kalisch-Ellett L, Caughey GE (2017). What is polypharmacy? A systematic review of definitions. BMC Geriatrics.

[4] Falster MO, Charrier R, Pearson SA, Buckley NA, Daniels B (2021). Long-term trajectories of medicine use among older adults experiencing polypharmacy in Australia. British Journal of Clinical Pharmacology.

[5] Wastesson JW, Morin L, Tan ECK, Johnell K (2018). An update on the clinical consequences of polypharmacy in older adults: A narrative review. Expert Opinion on Drug Safety.

[6] Tan ECK, Sluggett JK, Johnell K, Onder G, Elseviers M, Morin L, Vetrano DL, Wastesson JW, Fastbom J, Taipale H, Tanskanen A, Bell JS (2018). Research priorities for optimizing geriatric pharmacotherapy: An international consensus. Journal of the American Medical Directors Association.

[7] Smith SM, Wallace E, O'Dowd T, Fortin M (2016). Interventions for improving outcomes in patients with multimorbidity in primary care and community settings. Cochrane Database Systematic Review.

[8] Tinetti ME, Naik AD, Dodson JA (2016). Moving from disease-centered to patient goals-directed care for patients with multiple chronic conditions: Patient value-based care. JAMA Cardiology.

[9] Liau SJ, Lalic S, Sluggett JK, Cesari M, Onder G, Vetrano DL, Morin L, Hartikainen S, Hamina A, Johnell K, Tan ECK, Visvanathan R, Bell JS (2021). Medication management in frail older people: Consensus principles for clinical practice, research, and education. Journal of the American Medical Directors Association.

[10] Khanna D, Tsevat J (2007). Health-related quality of life–an introduction. The American Journal of Managed Care.

[11] Hand C (2016). Measuring health-related quality of life in adults with chronic conditions in primary care settings: Critical review of concepts and 3 tools. Canadian Family Physician.

[12] Wodchis WP, Hirdes JP, Feeny DH (2003). Health-related quality of life measure based on the minimum data set. International Journal of Technology Assessment in Health Care.

[13] Zubritsky C, Abbott KM, Hirschman KB, Bowles KH, Foust JB, Naylor MD (2013). Health-related quality of life: Expanding a conceptual framework to include older adults who receive long-term services and supports. The Gerontologist.

[14] Machón M, Larrañaga I, Dorronsoro M, Vrotsou K, Vergara I (2017). Health-related quality of life and associated factors in functionally independent older people. BMC Geriatrics.

[15] König HH, Heider D, Lehnert T, Riedel-Heller SG, Angermeyer MC, Matschinger H, Vilagut G, Bruffaerts R, Haro JM, de Girolamo G, de Graaf R, Kovess V, Alonso J (2010). Health status of the advanced elderly in six European countries: Results from a representative survey using EQ-5D and SF-12. Health and Quality of Life Outcomes.

[16] Andersson LB, Marcusson J, Wressle E (2014). Health-related quality of life and activities of daily living in 85-year-olds in Sweden. Health and Social Care in the Community.

[17] Sun W, Aodeng S, Tanimoto Y, Watanabe M, Han J, Wang B, Yu L, Kono K (2015). Quality of life (QOL) of the community-dwelling elderly and associated factors: A population-based study in urban areas of China. Archives of Gerontology and Geriatrics.

[18] Morin L, Johnell K, Laroche M-L, Fastbom J, Wastesson JW (2018). The epidemiology of polypharmacy in older adults: Register-based prospective cohort study. Clinical epidemiology.

[19] Charlesworth CJ, Smit E, Lee DS, Alramadhan F, Odden MC (2015). Polypharmacy Among Adults Aged 65 Years and Older in the United States: 1988–2010. Journals of Gerontology Series A.

[20] Rawle MJ, Richards M, Davis D, Kuh D (2018). The prevalence and determinants of polypharmacy at age 69: A British birth cohort study. BMC Geriatrics.

[21] Montiel-Luque A, Núñez-Montenegro AJ, Martín-Aurioles E, Canca-Sánchez JC, Toro-Toro MC, González-Correa JA (2017). Medication-related factors associated with health-related quality of life in patients older than 65 years with polypharmacy. PLoS ONE.

[22] Salinas-Rodríguez A, Manrique-Espinoza B, Rivera-Almaraz A, Ávila-Funes JA (2020). Polypharmacy is associated with multiple health-related outcomes in Mexican community-dwelling older adults. Salud Publica de Mexico.

[23] Lalic S, Jamsen KM, Wimmer BC, Tan ECK, Hilmer SN, Robson L, Emery T, Bell JS (2016). Polypharmacy and medication regimen complexity as factors associated with staff informant rated quality of life in residents of aged care facilities: A cross-sectional study. European Journal of Clinical Pharmacology.

[24] Vyas A, Alghaith G, Hufstader-Gabriel M (2020). Psychotropic polypharmacy and its association with health-related quality of life among cancer survivors in the USA: A population-level analysis. Quality of Life Research.

[25] Schenker Y, Park SY, Jeong K, Pruskowski J, Kavalieratos D, Resick J, Abernethy A, Kutner JS (2019). Associations between polypharmacy, symptom burden, and quality of life in patients with advanced, life-limiting Illness. Journal of General Internal Medicine.

[26] Watson N, Wooden M (2012). The HILDA survey: A case study in the design and development of a successful household panel survey. Longitudinal and Life Course Studies.

[27] Mellish L, Karanges EA, Litchfield MJ, Schaffer AL, Blanch B, Daniels BJ, Segrave A, Pearson SA (2015). The Australian pharmaceutical benefits scheme data collection: A practical guide for researchers. BMC Research Notes.

[28] Butterworth P, Crosier T (2004). The validity of the SF-36 in an Australian National Household Survey: Demonstrating the applicability of the Household Income and Labour Dynamics in Australia (HILDA) Survey to examination of health inequalities. BMC Public Health.

[29] Ware JE, Gandek B (1998). Overview of the SF-36 health survey and the international quality of life assessment (IQOLA) project. Journal of Clinical Epidemiology.

[30] Dockery M, Research, C. U. C. F. L. M (2006). Mental health and labour force status: panel estimates with four waves of HILDA.

[31] Wang L, Palmer AJ, Cocker F, Sanderson K (2017). Multimorbidity and health-related quality of life (HRQoL) in a nationally representative population sample: Implications of count versus cluster method for defining multimorbidity on HRQoL. Health and Quality of Life Outcomes.

[32] Australian Bureau of Statistics. Socio-economic indexes for areas (SEIFA). (2016). Technical Paper Retrieved Sep. 18 2020, from https://www.ausstats.abs.gov.au/ausstats/subscriber.nsf/0/756EE3DBEFA869EFCA258259000BA746/$File/SEIFA%202016%20Technical%20Paper.pdf

[33] Tegegn HG, Erku DA, Sebsibe G, Gizaw B, Seifu D, Tigabe M, Belachew SA, Ayele AA (2019). Medication-related quality of life among Ethiopian elderly patients with polypharmacy: A cross-sectional study in an Ethiopia university hospital. PLoS ONE.

[34] Vyas A, Kang F, Barbour M (2020). Association between polypharmacy and health-related quality of life among US adults with cardiometabolic risk factors. Quality of Life Research.

[35] Spoorenberg SLW, Wynia K, Uittenbroek RJ, Kremer HPH, Reijneveld SA (2018). Effects of a population-based, person-centred and integrated care service on health, wellbeing and self-management of community-living older adults: A randomised controlled trial on Embrace. PLoS ONE.

[36] Maruish, M. E. (2011). User's manual for the SF-36v2 Health Survey: Quality Metric Incorporated.

[37] Bjorner JB, Lyng Wolden M, Gundgaard J, Miller KA (2013). Benchmarks for interpretation of score differences on the SF-36 health survey for patients with diabetes. Value Health.

[38] Hilmer S, Gnjidic D (2009). The effects of polypharmacy in older adults. Clinical Pharmacology & Therapeutics.

[39] Budnitz DS, Pollock DA, Weidenbach KN, Mendelsohn AB, Schroeder TJ, Annest JL (2006). National surveillance of emergency department visits for outpatient adverse drug events. JAMA.

[40] Burt J, Elmore N, Campbell SM, Rodgers S, Avery AJ, Payne RA (2018). Developing a measure of polypharmacy appropriateness in primary care: Systematic review and expert consensus study. BMC Medicine.

[41] Rankin A, Cadogan CA, Patterson SM, Kerse N, Cardwell CR, Bradley MC, Ryan C, Hughes C (2018). Interventions to improve the appropriate use of polypharmacy for older people. The Cochrane Database of Systematic Reviews.

[42] World Health Organization (2019). Medication safety in polypharmacy: Technical report.

[43] Blum MR, Sallevelt BTGM, Spinewine A, O’Mahony D, Moutzouri E, Feller M, Baumgartner C, Roumet M, Jungo KT, Schwab N, Bretagne L, Beglinger S, Aubert CE, Wilting I, Thevelin S, Murphy K, Huibers CJA, Drenth-van Maanen AC, Boland B, Crowley E, Eichenberger A, Meulendijk M, Jennings E, Adam L, Roos MJ, Gleeson L, Shen Z, Marien S, Meinders A-J, Baretella O, Netzer S, de Montmollin M, Fournier A, Mouzon A, O’Mahony C, Aujesky D, Mavridis D, Byrne S, Jansen PAF, Schwenkglenks M, Spruit M, Dalleur O, Knol W, Trelle S, Rodondi N (2021). Optimizing Therapy to Prevent Avoidable Hospital Admissions in Multimorbid Older Adults (OPERAM): cluster randomised controlled trial. BMJ.

