# Supplementary material for: Polypharmacy and trajectories of health-related quality of life in older adults: an Australian cohort study
Source: Qual Life Res. 2022 Apr 27;31(9):2663–71. doi: 10.1007/s11136-022-03136-9 (PMC9356923; doi:10.1007/s11136-022-03136-9)
Supplement: Supplementary file 1 — Supplementary file1 (DOCX 128 kb) [file 11136_2022_3136_MOESM1_ESM.docx]

**Supplementary materials**
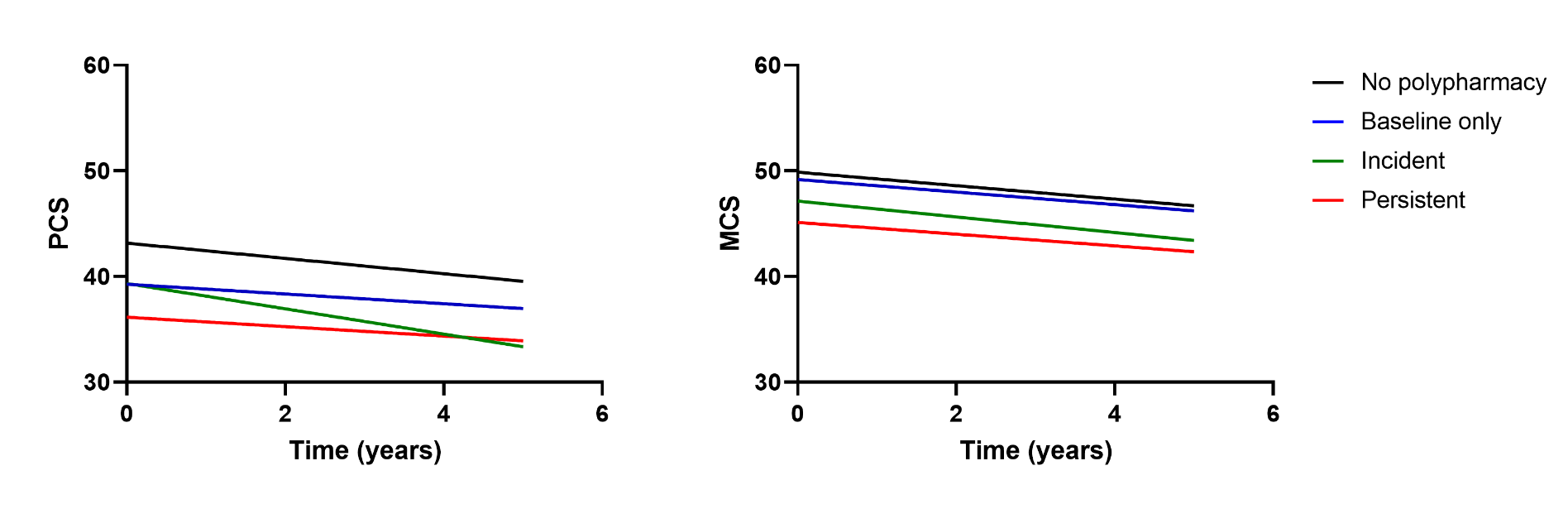


**Figure S1**. Quality of life trajectories in older adults with increase in comorbidities by polypharmacy strata. PCS: physical component summary; MCS: mental component summary.

Table S1: Available summary measure scores of health-related quality of life over the study period (2013 – 2017) for included participants

|  | N | Mean | SD |
| --- | --- | --- | --- |
| PCS 2013 | 2113 | 43.75 | 12.68 |
| MCS 2013 | 2122 | 50.81 | 11.55 |
| PCS 2014 | 2062 | 43.31 | 12.82 |
| MCS 2014 | 2067 | 50.56 | 11.70 |
| PCS 2015 | 2001 | 43.09 | 12.69 |
| MCS 2015 | 2004 | 50.46 | 11.90 |
| PCS 2016 | 1999 | 42.62 | 12.89 |
| MCS 2016 | 2003 | 50.21 | 11.84 |
| PCS 2017 | 1952 | 42.52 | 12.94 |
| MCS 2017 | 1954 | 50.24 | 11.91 |

PCS: Physical component summary; MCS: Mental component summary; SD: standard deviation

Table S2: Prevalence of chronic diseases by polypharmacy status at baseline

|  | Polypharmacy status | | | | |
| --- | --- | --- | --- | --- | --- |
|  | **Overall (n = 2165)*, n (%)** | **No polypharmacy (n = 1182), n (%)** | **Baseline only polypharmacy (n = 310), n (%)** | **Incident polypharmacy (n= 223), n (%)** | **Persistent polypharmacy (n= 450), n (%)** |
| Heart disease | 415 (19.2%) | 100 (8.5%) | 110 (35.5%) | 39 (17.5%) | 168 (36.9%) |
| Hypertension | 1342 (62.0%) | 663 (56.1%) | 198 (63.9%) | 137 (61.4%) | 344 (76.4%) |
| Any other serious circulatory condition | 209 (9.7%) | 44 (3.7%) | 59 (19.0%) | 24 (10.8%) | 82 (18.2%) |
| Arthritis or osteoporosis | 1229 (56.8%) | 622 (52.6%) | 202 (65.2%) | 114 (51.1%) | 291 (64.7%) |
| Type 1 diabetes | 44 (2.0%) | 13 (1.1%) | 10 (3.2%) | 3 (1.3%) | 18 (4.0%) |
| Type 2 diabetes | 355 (16.4%) | 100 (8.5%) | 77 (24.8%) | 36 (16.1%) | 142 (31.6%) |
| Asthma | 277 (12.8%) | 108 (9.1%) | 49 (15.8%) | 23 (10.3%) | 97 (21.6%) |
| Chronic bronchitis or emphysema | 148 (6.8%) | 45 (3.8%) | 31 (10.0%) | 15 (6.7%) | 57 (12.7%) |
| Depression or anxiety | 290 (13.4%) | 104 (8.8%) | 55 (17.7%) | 25 (11.2%) | 106 (23.6%) |
| Other mental illness | 24 (1.1%) | 6 (0.5%) | 7 (2.3%) | 2 (0.9%) | 9 (2.0%) |
| Any type of cancer | 229 (10.6%) | 115 (9.7%) | 40 (12.9%) | 20 (9.0%) | 54 (12.0%) |

*16 participants did not report having any chronic diseases

Table S3: Parameter estimates of mixed models for mental component summary (MCS) fitted in four polypharmacy strata for people with increase in comorbidities over four years

|  | *No polypharmacy,*  *N= 436* | | | Baseline only polypharmacy,  *N= 38* | | | *Incident polypharmacy,*  *N= 115* | | | *Persistent polypharmacy,*  *N= 178* | | |
| --- | --- | --- | --- | --- | --- | --- | --- | --- | --- | --- | --- | --- |
| **FIXED EFFECTS** | β (SE) | P value | 95% CI | β (SE) | P value | 95% CI | β (SE) | P value | 95% CI | β (SE) | P value | 95% CI |
| Intercept | 49.9 (0.7) | <0.001 | 48.5 ; 51.3 | 49.2 (1.5) | <0.001 | 46.1 ; 52.2 | 47.1 (1.0) | <0.001 | 45.2 ; 49.1 | 45.1 (0.9) | <0.001 | 43.3 ; 46.9 |
| Slope  (per year) | -0.6 (0.1) | <0.001 | -0.9 ; -0.4 | -0.6 (0.3) | 0.040 | -1.2 ; -0.03 | -0.8 (0.2) | <0.001 | -1.1 ; -0.4 | -0.6 (0.2) | 0.005 | -0.94 ; -0.17 |
| **RANDOM EFFECTS** |  |  |  |  |  |  |  |  |  |  |  |  |
| Variance in intercept | 63.6 (5.0) | <0.001 | 54.5 ; 74.2 | 47.3 (12.5) | <0.001 | 28.2 ;79.5 | 79.9 (11.8) | <0.001 | 59.8 ; 106.6 | 89.4 (10.9) | <0.001 | 70.44 ; 113.48 |
| Variance in slope | 0.9 (0.2) | <0.001 | 0.5 ; 1.5 | NA | NA | NA | 0.2 (0.5) | 0.755 | 0.0003 ; 84.2 | 1.9 (0.5) | <0.001 | 1.08 ; 3.29 |

Intercepts and slopes for all models are adjusted for age and sex. covariates were mean centred and included as intercept and slope predictors; MCS: Mental component summary; Polypharmacy defined as ≥5 medications.

Table S4: Parameter estimates of mixed models for physical component summary (PCS) fitted in four polypharmacy strata for people with increase in comorbidities over four years

|  | *No polypharmacy,*  *N= 436* | | | Baseline only polypharmacy,  *N= 38* | | | *Incident polypharmacy,*  *N= 115* | | | *Persistent polypharmacy,*  *N= 177* | | |
| --- | --- | --- | --- | --- | --- | --- | --- | --- | --- | --- | --- | --- |
| **FIXED EFFECTS** | β (SE) | P value | 95% CI | β (SE) | P value | 95% CI | β (SE) | P value | 95% CI | β (SE) | P value | 95% CI |
| Intercept | 43.2 (0.7) | <0.001 | 41.7 ; 44.6 | 39.3 (1.8) | <0.001 | 35.7 ; 42.9 | 39.3 (1.0) | <0.001 | 37.4 ; 41.3 | 36.2 (0.9) | <0.001 | 34.4 ; 37.9 |
| Slope  (per year) | -0.7 (0.2) | <0.001 | -1.03 ; -0.4 | -0.5 (0.4) | 0.194 | -1.2 ; 0.3 | -1.2 (0.2) | <0.001 | -1.6 ; -0.8 | -0.5 (0.2) | 0.010 | -0.8 ; -0.1 |
| **RANDOM EFFECTS** |  |  |  |  |  |  |  |  |  |  |  |  |
| Variance in intercept | 70.3 (0.3) | <0.001 | 60.2 ; 82.0 | 63.4 (18.6) | <0.001 | 35.8 ; 112.5 | 72.5 (10.9) | <0.001 | 54.1 ; 97.2 | 78.9 (9.4) | <0.001 | 62.5 ; 99.6 |
| Variance in slope | 1.3 (0.3) | <0.001 | 0.8 ; 2.0 | 0.3 (0.7) | 0.659 | 0.004 ; 27.6 | 0.1 (0.5) | 0.799 | 5.9 ; 274.7 | 0.3 (0.4) | 0.480 | 0.02 ; 4.6 |

Intercepts and slopes for all models are adjusted for age and sex only; All covariates were mean centred and included as intercept and slope predictors; PCS: physical component summary; Polypharmacy defined as ≥5 medications.

Table S5: Parameter estimates of mixed models for mental component summary (MCS) fitted in four polypharmacy strata for people with stable/decrease in comorbidities over four years adjusted for age and sex only

|  | *No polypharmacy,*  *N= 914* | | | Baseline only polypharmacy*,*  *N= 87* | | | *Incident polypharmacy, N= 130* | | | *Persistent polypharmacy,*  *N= 280* | | |
| --- | --- | --- | --- | --- | --- | --- | --- | --- | --- | --- | --- | --- |
| **FIXED EFFECTS** | β (SE) | P value | 95% CI | β (SE) | P value | 95% CI | β (SE) | P value | 95% CI | β (SE) | P value | 95% CI |
| Intercept | 54.02 | <0.001 | 53.4 ; 54.6 | 46.6 (1.3) | <0.001 | 44.02 ; 49.1 | 50.6 (0.8) | <0.001 | 48.9 ; 52.2 | 42.6 (0.7) | <0.001 | 41.2 ; 44.0 |
| Slope  (per year) | -0.01 (0.1) | 0.855 | -0.1 ; 0.1 | -0.02 (0.2) | 0.927 | -0.4 ; 0.4 | -0.8 (0.2) | <0.001 | -1.2 ; -0.4 | -0.1 (0.1) | 0.428 | -0.4 ; 0.2 |
| **RANDOM EFFECTS** |  |  | |  |  |  |  |  |  |  |  |  |
| Variance in intercept | 63.3 (3.3) | <0.001 | 57.1 ; 70.1 | 117.2 (19.6) | <0.001 | 84.5 ; 162.6 | 73.1 (10.6) | <0.001 | 55.1 ; 97.1 | 114.7 (10.8) | <0.001 | 95.4 ; 137.8 |
| Variance in slope | 0.3 (0.1) | 0.014 | 0.1 ; 0.7 | 0.3 (0.7) | 0.638 | 0.01 ; 20.1 | 1.8 (0.6) | 0.003 | 0.9 ; 3.5 | 1.0 (0.4) | 0.014 | 0.5 ; 2.2 |

Intercepts and slopes for all models are adjusted for age and sex. covariates were mean centred and included as intercept and slope predictors; MCS: Mental component summary; Polypharmacy defined as ≥5 medications.

Table S6: Parameter estimates of mixed models for mental component summary (MCS) fitted in four polypharmacy strata for people with increase in comorbidities over four years adjusted for age and sex only

|  | *No polypharmacy,*  *N= 436* | | | Baseline only polypharmacy*,*  *N= 38* | | | *Incident polypharmacy,*  *N= 115* | | | *Persistent polypharmacy,*  *N= 178* | | |
| --- | --- | --- | --- | --- | --- | --- | --- | --- | --- | --- | --- | --- |
| **FIXED EFFECTS** | β (SE) | P value | 95% CI | β (SE) | P value | 95% CI | β (SE) | P value | 95% CI | β (SE) | P value | 95% CI |
| Intercept | 53.4 (0.5) | <0.001 | 52.4 ; 54.4 | 48.1 (1.7) | <0.001 | 44.6 ; 51.6 | 48.1 | <0.001 | 46.1 ; 50.1 | 42.9 (0.9) | <0.001 | 41.1 ; 44.6 |
| Slope  (per year) | -0.6 (0.1) | <0.001 | -0.8 ; -0.4 | -0.3 (0.3) | 0.306 | -0.9 ; 0.3 | -0.8 (1.2) | <0.001 | -1.2 ; -0.5 | -0.5 (0.2) | 0.002 | -0.9 ; -0.2 |
| **RANDOM EFFECTS** |  |  |  |  |  |  |  |  |  |  |  |  |
| Variance in intercept | 78.7 (6.0) | <0.001 | 67.7 ; 91.4 | 78.2 (20.7) | <0.001 | 46.5 ; 131.4 | 96.5 (14.0) | <0.001 | 72.6 ; 128.4 | 119.9 (14.1) | <0.001 | 95.2 ; 151.1 |
| Variance in slope | 0.9 (0.2) | <0.001 | 0.5 ; 1.5 | 0.1 (0.6) | 0.902 | NA | 0.3 (0.6) | 0.533 | 0.01 ; 7.9 | 2.0 (0.6) | <0.001 | 1.1 ; 3.4 |

Intercepts and slopes for all models are adjusted for age and sex. covariates were mean centred and included as intercept and slope predictors; MCS: Mental component summary; Polypharmacy defined as ≥5 medications.

Table S7: Parameter estimates of mixed models for physical component summary (PCS) fitted in four polypharmacy strata for people with stable/decrease in comorbidities over four years adjusted for age and sex only

|  | *No polypharmacy,*  *N= 913* | | | Baseline only polypharmacy*,*  *N= 87* | | | *Incident polypharmacy,*  *N= 130* | | | *Persistent polypharmacy,*  *N= 280* | | |
| --- | --- | --- | --- | --- | --- | --- | --- | --- | --- | --- | --- | --- |
| **FIXED EFFECTS** | β (SE) | P value | 95% CI | β (SE) | P value | 95% CI | β (SE) | P value | 95% CI | β (SE) | P value | 95% CI |
| Intercept | 47.6 (0.4) | <0.001 | 46.9 ; 48.3 | 38.5 (1.2) | <0.001 | 36.2 ; 40.8 | 41.1 (1.0) | <0.001 | 39.1 ; 43.0 | 33.2 (0.7) | <0.001 | 31.9 ; 34.5 |
| Slope  (per year) | -0.2 (0.1) | 0.001 | -0.3 ; -0.1 | 0.01 (0.2) | 0.974 | -0.4 ; 0.4 | -0.8 (0.2) | <0.001 | -1.2 ; -0.4 | -0.2 (0.1) | 0.086 | -0.4 ; 0.03 |
| **RANDOM EFFECTS** |  |  |  |  |  |  |  |  |  |  |  |  |
| Variance in intercept | 79.2 (4.1) | <0.001 | 0.5 ; 1.2 | 97.4 (16.1) | <0.001 | 70.4 ; 134.8 | 106.7 (14.9) | <0.001 | 81.1 ; 140.4 | 99.1 (9.1) | <0.001 | 82.8 ; 118.7 |
| Variance in slope | 0.8 (0.2) | <0.001 | 0.5 ; 1.2 | 0.7 (0.5) | 0.222 | 0.1 ; 3.3 | 2.3 (0.7) | <0.001 | 1.3 ; 4.1 | 0.5 (0.3) | 0.098 | 0.2 ; 1.7 |

Intercepts and slopes for all models are adjusted for age and sex only; All covariates were mean centred and included as intercept and slope predictors; PCS: physical component summary; Polypharmacy defined as ≥5 medications.

|  | *No polypharmacy,*  *N= 436* | | | Baseline only polypharmacy,  *N= 38* | | | *Incident polypharmacy,*  *N= 115* | | | *Persistent polypharmacy,*  *N= 177* | | |
| --- | --- | --- | --- | --- | --- | --- | --- | --- | --- | --- | --- | --- |
| **FIXED EFFECTS** | β (SE) | P value | 95% CI | β (SE) | P value | 95% CI | β (SE) | P value | 95% CI | β (SE) | P value | 95% CI |
| Intercept | 47.3 (0.5) | <0.001 | 46.3 ; 48.3 | 38.4 (1.9) | <0.001 | 34.7 ; 42.2 | 40.8 (1.0) | <0.001 | 38.7 ; 42.8 | 33.7 (0.9) | <0.001 | 32.04 ; 35.4 |
| Slope  (per year) | -0.8 (0.1) | <0.001 | -1.03 ; -0.6 | -0.3 (0.4) | 0.414 | -1.02 ; 0.4 | -1.4 (1.0) | <0.001 | -1.7 ; -1.0 | -0.4 (0.2) | 0.008 | -0.7 ; -0.1 |
| **RANDOM EFFECTS** |  |  |  |  |  |  |  |  |  |  |  |  |
| Variance in intercept | 84.7 (6.5) | <0.001 | 72.9 ; 98.5 | 89.9 (25.5) | <0.001 | 51.6 ; 158.6 | 94.9 (14.1) | <0.001 | 70.9 ; 127.0 | 108.3 (12.5) | <0.001 | 86.4 ; 135.9 |
| Variance in slope | 1.3 (0.3) | <0.001 | 0.9 ; 2.0 | 1.1 (1.0) | 0.270 | 0.2 ; 6.4 | 0.7 (0.6) | 0.225 | 0.1 ; 3.6 | 0.4 (0.4) | 0.345 | 0.1 ; 3.3 |

Table S8: Parameter estimates of mixed models for physical component summary (PCS) fitted in four polypharmacy strata for people with increase in comorbidities over four years adjusted for age and sex only

Intercepts and slopes for all models are adjusted for age and sex only; All covariates were mean centred and included as intercept and slope predictors; PCS: physical component summary; Polypharmacy defined as ≥5 medications.

Table S9.1: Parameter estimates of incident polypharmacy and physical component summary (PCS) score in those with stable/decrease in comorbidities

| **Polypharmacy definition** | ≥3 medications  (n = 125) | | | ≥4 medications  (n = 139) | | | ≥6 medications  (n = 103) | | | ≥7 medications  (n = 86) | | |
| --- | --- | --- | --- | --- | --- | --- | --- | --- | --- | --- | --- | --- |
| **FIXED EFFECTS** | β (SE) | P value | 95% CI | β (SE) | P value | 95% CI | β (SE) | P value | 95% CI | β (SE) | P value | 95% CI |
| Intercept | 44.6 (0.9) | <0.001 | 42.8 ; 46.3 | 43.9 (0.9) | <0.001 | 42.2 ; 45.7 | 39.9 (1.1) | <0.001 | 37.8 ; 42.1 | 38.9 1.3 | <0.001 | 36.2 ; 41.5 |
| Slope  (per year) | -0.7 (0.2) | <0.001 | -1.0 ; -0.3 | -0.7 (0.2) | <0.001 | -1.1 ; -0.3 | -0.8 (0.2) | 0.001 | -1.3 ; -0.3 | -0.7 (0.3) | 0.015 | -1.3 ; -0.1 |
| **RANDOM EFFECTS** |  |  |  |  |  |  |  |  |  |  |  |  |
| Variance in intercept | 76.4 (10.8) | <0.001 | 57.8 ; 100.8 | 82.9 (11.4) | <0.001 | 63.3 ; 108.4 | 60.7 (9.9) | <0.001 | 44.1 ; 83.7 | 64.8 (11.4) | <0.001 | 46.0 ; 91.4 |
| Variance in slope | 0.8 (0.4) | 0.063 | 0.3 ; 2.3 | 0.8 (0.4) | 0.063 | 1.3 ; 3.5 | NA | NA | NA | NA | NA | NA |

Intercepts and slopes for all models are adjusted for age, sex, education, social life, marital status, Socio-Economic Indexes for Areas decile, physical activity, smoking and alcohol intake, and baseline comorbidities; All covariates were mean centred and included as intercept and slope predictors; PCS: physical component summary.

Table S9.2: Parameter estimates of incident polypharmacy and mental component summary (MCS) score in those with stable/decrease in comorbidities

| **Polypharmacy definition** | ≥3 medications  (n = 126) | | | ≥4 medications  (n = 140) | | | ≥6 medications  (n = 103) | | | ≥7 medications  (n = 86) | | |
| --- | --- | --- | --- | --- | --- | --- | --- | --- | --- | --- | --- | --- |
| **FIXED EFFECTS** | β (SE) | P value | 95% CI | β (SE) | P value | 95% CI | β (SE) | P value | 95% CI | β (SE) | P value | 95% CI |
| Intercept | 51.0  (0.8) | <0.001 | 49.4 ; 52.6 | 52.1 (0.7) | <0.001 | 50.7 ; 53.5 | 49.1 (1.1) | <0.001 | 46.9 ; 51.3 | 49.0 (1.4) | <0.001 | 46.2 ; 51.7 |
| Slope  (per year) | -0.6 (0.2) | <0.001 | -0.9 ; -0.3 | -0.5 (0.2) | 0.002 | -0.8 ; -0.2 | -0.6 (0.2) | 0.004 | -1.0 ; -0.2 | -0.8 (0.3) | 0.005 | -1.3 ; -0.2 |
| **RANDOM EFFECTS** |  |  |  |  |  |  |  |  |  |  |  |  |
| Variance in intercept | 63.5 (9.1) | <0.001 | 48.1 ; 84.0 | 54.0 (7.5) | <0.001 | 41.2 ; 70.9 | 79.6 (12.3) | <0.001 | 58.8 ; 107.8 | 80.4 (14.2) | <0.001 | 57.0 ; 113.5 |
| Variance in slope | 1.0 (0.5) | 0.036 | 0.4 ; 2.5 | 1.6 (0.4) | <0.001 | 0.9 ; 2.7 | NA | NA | NA | 0.6 (0.7) | 0.373 | 0.1 ; 5.4 |

Intercepts and slopes for all models are adjusted for age, sex, education, social life, marital status, Socio-Economic Indexes for Areas decile, physical activity, smoking and alcohol intake, and baseline comorbidities; All covariates were mean centred and included as intercept and intercept and slope predictors; MCS: Mental component summary.

Table S9.3: Parameter estimates of incident polypharmacy and physical component summary (PCS) score in those with increase in comorbidities

| **Polypharmacy definition** | ≥3 medications  (n = 134) | | | ≥4 medications  (n = 127) | | | ≥6 medications  (n = 104) | | | ≥7 medications  (n = 83) | | |
| --- | --- | --- | --- | --- | --- | --- | --- | --- | --- | --- | --- | --- |
| **FIXED EFFECTS** | β (SE) | P value | 95% CI | β (SE) | P value | 95% CI | β (SE) | P value | 95% CI | β (SE) | P value | 95% CI |
| Intercept | 43.1 (1.3) | <0.001 | 40.5 ; 45.0 | 43.9 (1.2) | <0.001 | 41.6 ; 46.1 | 37.1 (1.1) | <0.001 | 34.9 ; 39.2 | 35.2 (1.3) | <0.001 | 32.6 ; 37.8 |
| Slope  (per year) | -1.2 (0.3) | <0.001 | -1.8 ; -0.7 | -1.9 (0.2) | <0.001 | -2.2 ; -1.2 | -1.2 (0.2) | <0.001 | -1.5 ; -0.9 | -0.9 (0.2) | <0.001 | -1.3 ; -0.5 |
| **RANDOM EFFECTS** |  |  |  |  |  |  |  |  |  |  |  |  |
| Variance in intercept | 75.2 (10.7) | <0.001 | 57.0 ; 99.3 | 78.4 (11.3) | <0.001 | 59.1 ; 104.1 | 91.4 (13.7) | <0.001 | 68.1 ; 122.7 | 97.6 (16.5) | <0.001 | 70.1 ; 136.0 |
| Variance in slope | 1.1 (0.5) | 0.044 | 0.4 ; 2.9 | 1.0 (0.5) | 0.075 | 0.3 ; 2.9 | NA | NA | NA | NA | NA | NA |

Intercepts and slopes for all models are adjusted for age, sex, education, social life, marital status, Socio-Economic Indexes for Areas decile, physical activity, smoking and alcohol intake, and baseline comorbidities; All covariates were mean centred and included as intercept and slope predictors; PCS: physical component summary.

Table S9.4: Parameter estimates of incident polypharmacy and mental component summary (MCS) score in those with increase in comorbidities

| **Polypharmacy definition** | ≥3 medications  (n = 134) | | | ≥4 medications  (n = 127) | | | ≥6 medications  (n = 105) | | | ≥7 medications  (n = 84) | | |
| --- | --- | --- | --- | --- | --- | --- | --- | --- | --- | --- | --- | --- |
| **FIXED EFFECTS** | β (SE) | P value | 95% CI | β (SE) | P value | 95% CI | β (SE) | P value | 95% CI | β (SE) | P value | 95% CI |
| Intercept | 51.7 (1.2) | <0.001 | 49.0 ; 53.9 | 51.4 (1.1) | <0.001 | 49.2 ; 53.5 | 45.7 (1.1) | <0.001 | 43.6 ; 47.9 | 45.3 (1.2) | <0.001 | 42.8 ; 47.7 |
| Slope  (per year) | -0.9 (0.3) | 0.001 | -1.4 ; -0.3 | -1.2 (0.2) | <0.001 | -1.6 ; -0.8 | -1.0 (0.2) | <0.001 | -1.4 ; -0.6 | -1.3 (0.3) | <0.001 | -1.8 ; -0.8 |
| **RANDOM EFFECTS** |  |  |  |  |  |  |  |  |  |  |  |  |
| Variance in intercept | 69.2 (9.8) | <0.001 | 52.5 ; 91.3 | 70.2 (9.8) | <0.001 | 53.4 ; 92.3 | 96.4 (14.6) | <0.001 | 71.6 ; 129.7 | 95.4 (16.4) | <0.001 | 68.1 ; 133.9 |
| Variance in slope | 0.7 (0.5) | 0.175 | 0.2 ; 3.0 | 0.6  (0.4) | 0.159 | 0.2 ; 2.5 | NA | NA | NA | 1.9 (0.7) | 0.007 | 0.9 ; 3.9 |

Intercepts and slopes for all models are adjusted for age, sex, education, social life, marital status, Socio-Economic Indexes for Areas decile, physical activity, smoking and alcohol intake, and baseline comorbidities; All covariates were mean centred and included as intercept and slope predictors; MCS: Mental component summary

Table S10: Parameter estimates of mixed models for polypharmacy status, change in comorbidities and mental component summary (MCS) score over four years showing covariate estimates

| MCS |  |  |  |  |  |  |  |  |
| --- | --- | --- | --- | --- | --- | --- | --- | --- |
|  | *No polypharmacy, stable/decrease in comorbidities*  *N= 914* | *No polypharmacy, increase in comorbidities*  *N= 436* | Baseline only polypharmacy*, stable/decrease in comorbidities*  *N= 87* | Baseline only polypharmacy*, increase in comorbidities*  *N= 38* | *Incident polypharmacy, stable/decrease in comorbidities*  *N= 130* | *Incident polypharmacy, increase in comorbidities*  *N= 115* | *Persistent polypharmacy, stable/decrease in comorbidities*  *N= 280* | *Persistent polypharmacy, increase in comorbidities*  *N= 178* |
| Fixed effects |  |  |  | | | | | |
| *Intercept* | 54.0 (0.3) | 53.4 (0.5) | 46.6 (1.3) | 48.1 (1.7) | 50.6 (0.8) | 48.1 (1.0) | 42.6 (0.7) | 42.9 (0.9) |
| Age | -0.1 (0.1) | 0.1 (0.1) | 0.2 (0.2) | 0.4 (0.2) | 0.1 (0.1) | 0.2 (0.2) | 0.2 (0.1) | -0.03 (0.1) |
| Sex | -0.1 (0.6) | 0.1 (0.9) | 0.3 (3.1) | -2.4 (3.5) | 0.4 (1.7) | -2.9 (2.2) | 2.2 (1.3) | 3.3 (1.7) |
| comorbidtiy | -3.0 (0.3) | -3.1 (0.6) | -2.8 (1.3) | -3.1 (1.8) | -2.5 (0.8) | -2.6 (0.9) | -2.6 (0.5) | -2.1 (0.6) |
| Marital status | 2.8 (0.7) | 2.8 (1.2) | 4.5 (2.9) | 8.7 (3.6) | 2.4 (1.9) | 0.4 (2.5) | 1.3 (1.6) | 8.0 (2.0) |
| Social life | 0.4 (0.6) | 1.5 (1.0) | 0.7 (3.2) | -6.5 (3.2) | -0.7 (1.7) | 0.2 (2.3) | 3.5 (1.5) | 1.9 (2.0) |
| Alcohol | 3.8 (0.5) | 3.8 (0.9) | 6.2 (2.7) | 1.1 (3.01) | 2.0 (1.5) | 3.2 (2.1) | 7.5 (1.4) | 5.9 (1.7) |
| Physical status | -3.4 (1.2) | -6.5 (1.8) | -0.1 (5.7) | 5.4 (7.1) | -0.5 (3.4) | -0.3 (3.2) | 0.5 (2.9) | -1.4 (2.9) |
| Smoking | -0.1 (0.1) | 0.1 (0.1) | 0.2 (0.2) | 0.4 (0.2) | 0.1 (0.1) | 0.2 (0.2) | 0.2 (0.1) | -0.03 (0.1) |
|  | | | | | | | | |
| *Slope* |  | | | | | | | |
| Time | -0.01 (0.1) | -0.6 (0.1) | -0.02 (0.2) | -0.3 (0.3) | -0.8 (0.2) | -0.8 (0.2) | -0.1 (0.1) | -0.6 (0.2) |
| Age | -0.04 (0.01) | -0.1 (0.02) | -0.1 (0.03) | -0.02 (0.1) | -0.1 (0.03) | -0.1 (0.03) | -0.2 (0.02) | 0.002 (0.03) |
| Sex | -0.2 (0.1) | -0.3 (0.2) | -1.2 (0.5) | 2.3 (0.8) | -0.3 (0.4) | 0.4 (0.4) | -0.3 (0.3) | -0.1 (0.4) |
| Comorbidities | 0.03 (0.1) | -0.1 (0.1) | 0.3 (0.2) | -0.4 (0.4) | -0.2 (0.2) | 0.1 (0.2) | -0.1 (0.1) | -0.1 (0.1) |
| Marital status | -0.1 (0.1) | -0.02 (0.2) | -1.0 (0.5) | -0.2 (0.7) | -0.6 (0.4) | -0.1 (0.4) | -0.5 (0.3) | 0.3 (0.4) |
| Social life | -0.2 (0.2) | -0.2 (0.2) | -0.9 (0.5) | -1.4 (0.9) | -0.02 (0.5) | 0.6 (0.5) | 0.03 (0.3) | -0.5 (0.4) |
| Alcohol | 0.1 (0.1) | -0.04 (0.2) | -0.3 (0.5) | 0.3 (0.7) | 0.6 (0.4) | 0.1 (0.4) | -0.7 (0.3) | -0.4 (0.4) |
| Physical status | -0.1 (0.1) | -0.2 (0.2) | -0.5 (0.5) | 0.9 (0.7) | 0.5 (0.4) | 0.2 (0.4) | -0.3 (0.3) | -0.3 (0.4) |
| Smoking | 0.1 (0.2) | -0.1 (0.4) | 0.6 (0.9) | -0.7 (1.5) | 0.1 (0.9) | -1.02 (0.6) | -0.4 (0.6) | -0.6 (0.7) |

Intercepts and slopes for all models are adjusted for age, sex, education, social life, marital status, Socio-Economic Indexes for Areas decile, physical activity, smoking and alcohol intake, and baseline comorbidities; All covariates were mean centred and included as intercept and slope predictors; PCS: physical component summary; Polypharmacy defined as ≥5 medications.

Table S11: Parameter estimates of mixed models for polypharmacy status, change in comorbidities and physical component summary (PCS) score over four years showing covariate estimates

| PCS |  |  |  |  |  |  |  |  |
| --- | --- | --- | --- | --- | --- | --- | --- | --- |
|  | *No polypharmacy, stable/decrease in comorbidities*  *N= 913* | *No polypharmacy, increase in comorbidities*  *N= 436* | Baseline only polypharmacy*, stable/decrease in comorbidities*  *N= 87* | Baseline only polypharmacy*, increase in comorbidities*  *N= 38* | *Incident polypharmacy, stable/decrease in comorbidities*  *N= 130* | *Incident polypharmacy, increase in comorbidities*  *N= 115* | *Persistent polypharmacy, stable/decrease in comorbidities*  *N= 280* | *Persistent polypharmacy, increase in comorbidities*  *N= 177* |
| Fixed effects |  | | | | | | | |
| *Intercept* | 45.3 (0.3) | 43.2 (0.7) | 40.9 (1.3) | 39.3 (1.8) | 42.5 (1.0) | 39.3 (1.0) | 38.7 (0.9) | 36.2 (0.9) |
| Age | -0.1 (0.1) | 0.1 (0.1) | 0.2 (0.2) | -0.1 (0.3) | -0.1 (0.1) | -0.04 (0.2) | -0.1 (0.1) | -0.2 (0.1) |
| Sex | -0.1 (0.6) | 0.1 (0.9) | 0.3 (3.1) | -6.4 (3.9) | -2.7 (1.9) | -1.0 (2.2) | -2.5 (1.2) | 1.3 (1.6) |
| comorbidtiy | -2.9 (0.3) | -3.1 (0.6) | -2.8 (1.3) | -1.7 (2.0) | -3.2 (0.9) | -3.5 (0.9) | -2.2 (0.4) | -2.3 (0.6) |
| Marital status | 1.01 (0.6) | 0.1 (1.0) | 3.5 (2.9) | -0.5 (3.7) | 4.1 (1.0) | -0.2 (2.1) | -0.1 (1.2) | 4.4 (1.6) |
| Social life | 2.8 (0.7) | 2.8 (1.3) | 4.5 (2.9) | 2.6 (4.0) | 2.6 (2.3) | -0.5 (2.5) | -0.01 (1.4) | 2.8 (1.9) |
| Alcohol | 0.4 (0.6) | 1.5 (0.9) | 0.7 (3.2) | -7.2 (3.5) | 0.5 (2.1) | 0.8 (2.3) | 2.3 (1.4) | 2.8 (1.9) |
| Physical status | 3.8 (0.5) | 3.8 (0.9) | 6.2 (2.7) | 4.9 (3.4) | 4.7 (1.8) | 4.9 (2.0) | 8.7 (1.3) | 5.8 (1.6) |
| Smoking | -3.4 (1.2) | -6.4 (1.8) | -0.1 (5.7) | -5.2 (8.0) | -2.8 (4.2) | 1.9 (3.1) | 1.3 (2.7) | 0.4 (2.8) |
|  | | | | | | | | |
| *Slope* |  | | | | | | | |
| Time | -0.2 (0.1) | -0.7 (0.2) | -0.3 (0.2) | -0.5 (0.4) | -0.9 (0.2) | -1.2 (0.2) | -0.2 (0.2) | -0.5 (0.2) |
| Age | -0.04 (0.01) | -0.1 (0.02) | -0.1 (0.03) | 0.02 (0.1) | -0.03 (0.03) | -0.02 (0.03) | -0.1 (0.02) | -0.01 (0.02) |
| Sex | -0.2 (0.1) | -0.3 (0.2) | -1.2 (0.5) | 2.5 (0.8) | 0.1 (0.4) | 0.3 (0.4) | 0.2 (0.2) | -0.1 (0.3) |
| Comorbidity | 0.03 (0.1) | -0.1 (0.1) | 0.3 (0.2) | -0.6 (0.4) | 0.1 (0.2) | 0.4 (0.2) | -0.1 (0.1) | 0.1 (0.1) |
| Marital status | -0.1 (0.1) | -0.02 (0.2) | -1.0 (0.5) | 0.4 (0.7) | -0.3 (0.4) | -0.2 (0.4) | -0.2 (0.2) | 0.1 (0.3) |
| Social life | -0.2 (0.2) | -0.2 (0.2) | -0.9 (0.5) | -0.3 (0.9) | -0.3 (0.5) | 0.4 (0.5) | 0.1 (0.3) | 0.3 (0.4) |
| Alcohol | 0.1 (0.1) | -0.04 (0.2) | -0.3 (0.5) | -0.2 (0.7) | 0.8 (0.5) | -0.5 (0.5) | -0.4 (0.3) | -0.4 (0.4) |
| Physical status | -0.1 (0.1) | -0.2 (0.2) | -0.5 (0.5) | 1.1 (0.7) | 0.3 (0.4) | 0.6 (0.4) | -0.8 (0.3) | -0.3 (0.3) |
| Smoking | 0.1 (0.2) | -0.1 (0.4) | 0.6 (0.9) | 1.2 (1.5) | 1.4 (0.9) | -1.3 (0.6) | -1.3 (0.5) | -0.9 (0.6) |

Intercepts and slopes for all models are adjusted for age, sex, education, social life, marital status, Socio-Economic Indexes for Areas decile, physical activity, smoking and alcohol intake, and baseline comorbidities; All covariates were mean centred and included as intercept and slope predictors; PCS: physical component summary; Polypharmacy defined as ≥5 medications.
